# Development of hop transcriptome to support research into host-viroid interactions

**DOI:** 10.1371/journal.pone.0184528

**Published:** 2017-09-08

**Authors:** Tine Pokorn, Sebastjan Radišek, Branka Javornik, Nataša Štajner, Jernej Jakše

**Affiliations:** 1 Agronomy Department, Biotechnical Faculty, University of Ljubljana, Ljubljana, Slovenia; 2 Department of Plant Protection, Slovenian Institute of Hop Research and Brewing, Žalec, Slovenia; Agriculture and Agri-Food Canada, CANADA

## Abstract

Viroids, the smallest known pathogens, unable to encode any proteins, can cause severe diseases in their host plants. One of the proposed mechanisms of their pathogenicity includes silencing the host’s genes via viroid-derived small RNAs, which are products of the host’s immune response to the viroid’s double stranded RNA. *Humulus lupulus* (hop) plants are hosts to several viroids; two of them, HLVd and CBCVd, are interesting models for studying host-viroid interactions, due to the symptomless infection of the former and severe stunting disease caused by the latter. To study these interactions, we constructed a deep hop NGS transcriptome based on 35 Gb paired-end sequencing data assembled into over 74 Mb of contigs. These transcripts were used for *in-silico* prediction of target transcripts of vd-sRNA of the two aforementioned viroids, using two different software tools. Prediction models revealed that 1062 and 1387 hop transcripts share nucleotide similarities with HLVd- and CBCVd-derived small RNAs, respectively, so they could be silenced in an RNA interference process. Furthermore, we selected 17 transcripts from 4 groups of targets involved in the metabolism of plant hormones, small RNA biogenesis, transcripts with high complementarity with viroid-derived small RNAs and transcripts targeted by CBCVd-derived small RNAs with high cellular concentrations. Their expression was monitored by reverse transcription quantitative PCR performed using leaf, flower and cone samples. Additionally, the expression of 5 pathogenesis related genes was monitored. Expression analysis confirmed high expression levels of four pathogenesis related genes in leaves of HLVd and CBCVd infected hop plants. Expression fluctuations were observed for the majority of targets, with possible evidence of downregulation of GATA transcription factor by CBCVd- and of linoleate 13S-lipoxygenase by HLVd-derived small RNAs. These results provide a deep transcriptome of hop and the first insights into complex viroid-hop plant interactions.

## Introduction

Viroids are the smallest known plant pathogens, consisting of a single-stranded, covalently closed circular RNA molecule, which forms secondary structures. Current taxonomy (2015) of the International Committee on Taxonomy of Viruses (ICTV) [1] includes 32 confirmed viroid species, while NCBI’s taxonomy comprises 45 species, including as yet unclassified viroids. Their genome is smaller than an average plant transcript, usually in the size range from 246 to 412 bp, with an average size of 337.2 bp, and they do not encode for any protein or transcribed sequence. Phylogenetically, viroids are classified into two major groups: *Avsunviroidae* and *Pospiviroidae*. The four viroid species of the first taxonomic group are localized to the chloroplasts, with a rod-shape, branched structure, exhibit ribozyme-like catalytic activity and follow a symmetric pathway of replication. The most abundant viroids, the *Pospiviroidae* group, are localized to the nucleus, consist of rod-like secondary structures and follow an asymmetric pathway of replication [2].

Although they are such insignificant RNA molecules, viroids can cause considerable damage and economic losses in plant production agricultural systems. Probably the most well-known example is the lethal *Coconut cadang cadang viroid* (CCCVd), the presence of which almost eradicated coconut production in the Philippines, with up to half a million plants still dying back on an annual scale [3]. Potato crop and seed losses of up to 1% in North America due to *the Potato spindle tuber viroid* (PSTVd) should also be noted [3].

Hop (*Humulus lupulus* L.) is currently known to be host for three viroid species *Hop latent viroid* (HLVd), *Hop stunt viroid* (HSVd) and *Citrus bark cracking viroid* (CBCVd) and a taxonomically uncharacterized species of *Apple fruit crinkle viroid* (AFCVd). Infection caused by HLVd has been reported worldwide [4]. Plants are symptomless, although HLVd free hop plants have higher yields and higher alpha-acid content [5]. Reinfection of HLVd viroid free plants in field conditions occurs very rapidly in a few years [6]. HSVd was first discovered in Japanese hop fields, causing hop stunt disease, which appeared 3–5 years after infection as plant stunting, small cone formation and a substantial reduction of alpha-acid content [7,8]. The occurrence of this viroid, which is nowadays known to be commonly present in several woody hosts, including grapevine [9], was later also confirmed in other hop growing countries [10–12]. AFCVd symptoms on hop resemble those of HSVd, and it is currently restricted to Japan [13]. The latest viroid discovered on hop is CBCVd, which causes the most aggressive symptoms among all 4 hop viroids. Infected plants show the first symptoms 4 months after infection: severe stunting after first dormancy and complete dieback in 3–5 years [14,15].

Soon after the first viroid species discoveries [16], questions were raised about their ability to trigger severe symptoms in some plants and about the mechanisms of their action. Being plant pathogens with genomes of the smallest size and complexity, and well defined structures, it was thought that answers would quickly be found, but this puzzle is still challenging the viroid research community. There is growing experimental evidence that the model proposing attenuation of host gene expression by viroid-derived sRNAs (vd-sRNAs) via directed RNAi, actually takes place in viroid-host pathosystem models. The first evidence–the presence of vd-sRNAs in infected plants—was revealed by hybridization approaches [17–19]. Subsequently, the advent of NGS sequencing methodologies clearly confirmed an enormous accumulation of vd-sRNA for both taxonomic groups of viroids in infected plant tissues [20,21]. The second body of evidence supporting the role of RNAi mechanisms is the confirmed loading of PSTVd derived sRNAs in RISC components; namely, association with selected Argonaute (AGO) proteins, thus being capable of attenuating the levels of complementary targets [22]. The third body of evidence–similarities between vd-sRNAs and the plant’s transcripts and confirmed altered expressions of these targets—is also mounting. In a form of albinism called peach calico, caused by chloroplast-replicating *Peach latent mosaic viroid* (PLMVd), it was shown that two PLMVd-sRNAs originate from the (-) strand of calico strain target for cleavage of the chloroplast transcript of heat-shock protein 90 (cGSP90), which is in agreement with chloroplast abnormalities caused by the viroid [23]. Analysis of PSTVd variants causing different levels of symptoms in tomato enabled researchers to discover molecular and biological evidence that PTSVd-derived sRNAs target and silence at least two tomato callose synthase genes up to 1.5-fold and thus remove an important biological factor for the viroid’s cell-to-cell spread [24]. By using computational prediction of similarities between *Arabidopsis* genes and vd-sRNAs from 29 species of the *Pospiviroidae* family, a tomato’s conserved WD40-repeat protein was identified as a possible target of silencing [25].

Since plants are sessile living organisms, they develop sophisticated mechanisms of defence against biotic stress. During pathogen infection and disease progression, several defence pathways are triggered and mainly regulated by hormone signalling pathways, such as salicylic acid (SA), jasmonic acid (JA) and ethylene (ET) [26], including other phytohormone regulators, such as abscisic acid (ABA), brassionsteroids (BR), auxins (IAA), cytokinins (CK) and strigolactones [27]. In turn, pathogens have developed mechanisms to interfere with these signalling pathways in order to overcome the plant defence for successful infection and propagation. Bacterial, fungal, oomycete and viral pathogens mainly use a plethora of fast evolving protein effector molecules for these manipulations [28]. Viroids are unable to produce any of these effector proteins; it would therefore be intriguing to solve the enigma of whether they are also capable of hijacking these plant defence pathways. Developmental changes associated with some viroid caused diseases, such as stunting, leaf epinasty and alternation of flowering time, support the idea of impaired hormonal balance in viroid infected plants [29]. The latest evidence, obtained from plant-pathogen interactions, widens the effector repertoire to include a role of sRNA molecules, such as was confirmed in fungus *Botrytis cinerea* interactions with the plant host [30].

The aim of the present work was to investigate the role of two viroids of the *Pospiviroidae* family, with different development of symptoms in hop plants. Infection by HLVd is visually symptomless, while CBCVd causes severe visible disease symptoms and phenotypic changes. This research focused primarily on expression analysis of host genes, which were selected based on the predicted complementarity between vd-sRNA and the host transcript, their high cellular concentration and their involvement in hormonal pathways and biogenesis of small RNAs, based on a developed NGS hop transcriptome dataset. Since PR proteins are regarded as biomarkers of plant health status, we have also included expression analysis of selected pathogenesis related (PR) proteins to compare their upregulation in symptomless HLVd and severely affected CBCVd plants.

## Materials and methods

### Plant material

The hop variety ‘Celeia’ was chosen for sampling, since it is one of the most widely cultivated and CBCVd affected varieties in Slovenia. Sampling was performed in the production fields of the Slovenian Institute for Hop Research and Brewing (SIHRB) and in privately owned fields near Šempeter in the 2013 growing season. The hop fields were maintained under standard agricultural practice. If hop plants are not viroid free, they are always infected with HLVd, and natural CBCVd infection is also always accompanied by HLVd infection.

Samples for hop transcriptome construction were collected from eight CBCVd free plants (HLVd present) during the whole growing season, at 14 days intervals from the beginning of sprouting until harvest (end of March until end of August). Sampled tissues included roots, emerged shoots, leaves, stems, flowers and cones.

Leaves, flowers and cones from at least 5 different plants were collected for quantitative reverse transcription PCR (RT-qPCR), from 1) viroid free plants, 2) HLVd infected plants (CBCVd absent) and 3) CBCVd infected plants (HLVd also present), in which the viroid presence was confirmed by RT-PCR. Collected tissues samples were immediately put on dry ice and transferred to the laboratory, in which RNA was isolated. Tissue samples were also stored at -80°C.

### RNA isolation

Total RNA was extracted from collected plant material using a Spectrum^™^ Plant Total RNA Kit (Sigma-Aldrich) following the manufacturer’s protocol A, followed by an on-column DNase Digestion step. Eluted RNA samples were quantified by NanoVue spectrophotometer (GE Healthcare) readings of absorbance at 260 and 280 nm. RNA integrity and quality were established by formaldehyde gel electrophoresis and further by Bioinalayzer 2100 RNA integrity number (RIN) determination using an RNA 6000 Nano Kit (Agilent). Samples with RINs >7 were used for further NGS sequencing or RT-qPCR analysis. Isolated RNA samples were stored at -80°C.

### RT-PCR

Plant samples (viroid free, HLVd and CBCVd infected) were inspected for healthy status or viroid presence using reverse transcription PCR (RT-PCR). Plants were tested for the presence of HLVd and CBCVd using a OneStep RT-PCR Kit (Qiagen). Total RNA (1 ng) was mixed with two viroid specific primers to yield a final concentration of 0.6 μM: a) for CBCVd primers CVd-IV-F1 5'-GGGGAAATCTCTTCAGAC-3' and CVd-IV-R1 5'-GGGGATCCCTCTTCAGGT-3' [31] and b) for HLVd primers HLVd-M 5'-TAGTTTCCAACTCCGGCTGG-3' and HLVd-P 5'-GGATACAACTCTTGAGCGCC-3' were used [32]. Five μl of RNA and primer mixture was denatured at 95°C for 5 min and immediately chilled on ice. Components of the RT-PCR kit were subsequently added, yielding a total volume of 12 μl, with final concentrations as follows: 1x OneStep RT-PCR Buffer, 0.4 mM of each dNTP, 1x Q-Solution and 0.48 μl of OneStep RT-PCR Enzyme Mix. Reactions were incubated in a PE9700 thermal cycler with the following temperature profile: 50°C for 30 min for the reverse transcription step, followed by a PCR amplification step with initial denaturation at 95°C for 15 min, followed by 40 amplification cycles starting at 94°C 30 sec, 65°C 30 sec, 72°C 60 sec, whereby the annealing temperature was lowered by 1°C for the 9 subsequent cycles, then maintained at 56°C and amplification was finished by 10 min incubation at 72°C. Amplification of PCR products was confirmed by TBE electrophoresis on 1.5% agarose gels stained with ethidium bromide (0.5 μg/ml) and visualized on an UV source.

### Hop transcriptome NGS sequencing and analysis

Total RNA samples of whole season sampled ‘Celeia’ plants were pooled together in an equimolar amount to yield a final sample for sequencing, which was performed at IGA Technology Services Srl (Udine, Italy). The sample was rRNA depleted using a Ribo-Zero rRNA Removal Kit for Plants (Illumina). An RNA library (insert size 200–300 bp) was constructed following the protocol of the TruSeq RNA-seq Sample Preparation Kit (Illumina Inc.). Sequencing was performed on a single HiSeq2000 lane by a paired-end (PE) 100 bp set-up and raw sequences were delivered in FASTQ format. They were submitted to Sequence Read Archive (SRA) [33] for public availability.

NGS data were analysed with the CLC genomics Workbench or CLC Genomics Server tools (CLC). First sequencing data quality control (QC) was performed using the tool ‘Create Sequencing QC Report’. Quality, ambiguity and adapter sequence trimming was performed with the ‘Trim Sequences’ tool, using a limit of 0.05 for quality score, with the maximum number of allowed ambiguities set to 3 and the minimum length of clean reads to 30 bp. If one sequence of a pair was removed due to the set threshold, the other sequence was kept as a single end sequence and used in further analysis. The level of perfect duplicates was assessed with the aid of the python script process-reads-fasta.py, as part of the miR-PREFeR pipeline [34]. *De-novo* assembly of trimmed sequences was performed using the ‘*De-novo* assembly’ module of the CLC, using the following parameters: bubble size 50, word size 24 and minimum contig length 150 with the scaffolding option ‘on’, remapping ‘yes’.

The completeness of the obtained hop transcriptome was assessed with the BUSCO tool, which performs evolutionarily informed expectations of gene content from near-universal single-copy orthologs using the plant early release dataset version [35].

The resulting assembled contigs were searched against Genbank protein ‘nr’ and nucleotide ‘nt’, Swiss-Prot protein, and Eukaryotic Orthologous Groups of proteins (KOG) databases, using blastx or blastn algorithms of the standalone version of the blast+ tool [36]. Results were stored in BLAST archive format for further processing with the blast_formatter tool as needed.

The Blast2GO command line software tool [37] was used for automatic functional annotation of assembled transcripts to obtain the Gene Ontology (GO) terms, which describe biological processes, molecular function and cellular components.

### Prediction of vd-sRNA targets

RNA sequences of HLVd (GenBank X07397) and CBCVd (GenBank KM211546) viroids were *in-silico* cut to all possible (+) and (-) 21 bp, 22 bp and 24 bp mers representing vd-sRNAs, bearing in mind the circular nature of viroids. Small RNAs were searched for similarity against the hop transcriptome, using two tools designed to identify possible targets of sRNAs action with default parameters: 1) the UEA sRNA Toolkit [38], which was used as a standalone application (version May 18, 2012) and 2) the Plant Small RNA Target Analysis Server (psRNA Target) [39], which was used as a WWW application available at: http://plantgrn.noble.org/psRNATarget/.

In the selection of targets for RT-qPCR analysis, we focused on four different groups of hop genes: **1)** genes involved in the metabolism of plant hormones, e.g., ethylene, gibberellins, jasmonates and salicylic acid, due to their involvement in plant defence pathways against pathogens [40–43]. The list of *Arabidopsis* genes involved in these metabolic pathways was taken from the RIKEN Plant Hormone Research Network (http://hormones.psc.riken.jp/pathway_hormones.shtml) and corresponding protein sequences were used for tblastn search against the hop transcriptome, **2)** genes of the RNAi machinery, **3)** genes with complete or almost complete complementarity between the target and vd-sRNAs and **4)** targets of CBCVd derived sRNAs with high cellular concentrations, as revealed by NGS mapping profiles [14]. The coverage statistics were obtained from BAM files using samtools [44].

### Primer design

To avoid creating primers in the regions of conserved protein domains, all sequences were searched using NCBI’s CD-Search tool against the CDD database. Regions of the revealed domains were avoided for primer design where possible in order to increase amplification specificity. Primer Express 3.0 software (90 bp amplicon length, optimal T_m_ at 60°C, GC% between 30% and 80%) was used to design primers from selected targets of either HLVd or CBCVd derived sRNA action. DEAD box RNA helicase (DRH1), TIP41-like family protein (TIP41) and Yellow leaf specific protein 8 (YLS8) [45] primers were used for reference gene amplification. Hop specific primers for monitoring the expression of five pathogenesis related (PR) proteins were also used: PR-1, PR-2 (β-1,3-glucanase), PR-3-EX (extracellular type chitinase), PR-3-VAC (vacuole type chitinase), and PR-5 (thaumatin-like protein)[46].

### Two-step reverse transcription quantitative PCR (RT-qPCR) and data analysis

Single stranded cDNA was synthesized from isolated total RNA. One microgram of each RNA sample, represented by at least 3 biological replicates per experiment point, was reverse transcribed to cDNA with a High Capacity cDNA Reverse Transcription Kit (Applied Biosystems, Foster City, USA) using random hexamers. Real-time PCR in three technical replicates was performed using Fast SYBR Green Technology in the ABI PRISM 7500 Fast Sequence Detection System (Applied Biosystems, Foster City, USA). A master mix for each PCR run was prepared using a 10 μl reaction volume and containing 5 μl of FastStart Universal SYBR Green Master (Roche, Switzerland), 2 μl cDNA (corresponding to 5 ng of total RNA) and 300 nM (0.6 μl) of each primer on MicroAmp Optical 96-well PCR plates (Applied Biosystems, USA). The following amplification protocol was used: 95°C 10 min, 40 cycles at 95°C for 10 s followed by 60°C for 30 s. The specificity of amplification was confirmed by melting curve analysis. All the reactions with a single primer pair were performed on the same plate to minimize variation between runs and a standard curve included on each plate. The efficiency for each target was calculated using the slope of the regression line in the standard curve performed on bulked cDNA samples of five serial dilutions in a ratio 1:4 in a range from 12.5 to 0.05 ng.

Statistical analysis of RT-qPCR results was performed using the delta-delta-Ct method (ΔΔCt), which is the method of choice for analysing relative changes in gene expression [47]. Using this method, the data are presented as fold change values (FC) in gene expression normalized to the endogenous reference genes used (DRH1, TIP41 and YLS8), which were defined as optimal in previous hop biotic stress studies [45] and relative to the untreated control, which in our case were viroid free plants.

## Results

### NGS sequencing and de-novo assembly of the transcriptome

Illumina sequencing of ribosomal depleted hop RNA in a single lane yielded 348,065,384 reads in paired orientation, each 100 bp long, totalling over 35 Gb of raw transcriptome data. The number of unique reads when perfect duplicates were clustered in the set was 50,200,620, and 56,117,015 reads for each single file of the paired end data. The GC content of the raw sequencing data was estimated at 45.5%. The raw sequences were deposited in NCBI’s SRA archive for public availability under BioProject number PRJNA342762, BioSample SAMN05767836, SRA run SRR4242068.

Trimming, which included removing low quality and ambiguous regions, removal of adaptor sequences and removal of too-short reads, resulted in a total of 343,559,950 sequences (98.7% of total), of which 339,494,248 in paired-end orientation and 4,065,702 as single end sequences of an average read length of 97.2 bp, corresponding to about 33.4 Gb of cleaned sequence data. These were considered to be of high quality and were used for further analysis.

*De-novo* assembly with the scaffolding option and mapping reads back to contigs produced 150,443 contigs (including scaffolds), with a total length of the assembled transcriptome being over 74 Mbp, which represented a reference expressed whole plant transcriptome of *H*. *lupulus*. The number of sequences with a length ≥ 200 bp (length constraint for NCBI’s Transcriptome Shotgun Assembly archive) was 92,464, although all sequences were considered in further downstream analysis. The N50, N75 value and average length were calculated as 984 bp, 296 bp and 494 bp, respectively. The longest contig assembled was 23,284 bp. Since the *de-novo* assembly step used remapping of the reads, coverages for transcriptome contigs were calculated as 145X on average. Assembly metrics for the hop transcriptome are presented in Table 1.

**Table 1:** Hop transcriptome assembly metrics of Illumina paired end reads.

Number of contigs	162,889
Number of contigs including scaffolded regions	150,443
N50 contigs	743 bp
N50 contigs including scaffolded region	984 bp
Maximum length contigs	23,284 bp
Maximum length scaffolds	23,284 bp
Average length contigs	452 bp
Average length scaffolds	494 bp
Total length contigs	73,699,989 bp
Total length including scaffolded regions	74,308,957 bp
Total reads used in assembly	343,559,950
Matched reads	295,962,373 (86.1% of all)
Not matched reads	47,597,577 (13.9% of all)
Reads in pairs	245,947,392 (83.1% of all)
Maximum coverage	345,180X
Average coverage	145X
Coverage SD	±1918X

### Transcriptome annotation

The transcriptome completeness was assessed against 956 BUSCO groups using the BUSCO software package [35]. Comparison revealed it to be 78% complete, taking into account similarities of complete single-copy (553) and complete duplicated (194) BUSCOs, or 89% complete also taking into account 106 fragmented BUSCOs. Eleven percent (103) of BUSCOs were missing in the assembled hop transcriptome.

The transcriptome was further annotated by comparison of the *H*. *lupulus* transcriptome with several public sequence databases. BLASTx query against the NCBI nr database did not return any significant protein hits for 106,798 sequences (71%). Sequences without hits belonged to the group of shorter sequences with an average length of 284 bp and with quartile 2 value of 206 bp. The remaining 43,465 sequences, with BLAST hits, had significantly (t-test, p<0.01) longer average length and quartile 2 value of 1006 bp and 512 bp, respectively. Similar results were obtained with searches against Swiss-Prot and KOG protein databases, whereby 116,951 and 109,172 *H*. *lupulus* transcripts, respectively, did not reveal any significant hits. This is evident from BLASTn searches of transcripts against NCBI’s nt database, in which a lower number of transcripts (88,101; 58.56%) remained unannotated. The final percentage of all hop transcripts annotated with the BLAST comparison was 54.83% (Table 2).

**Table 2:** BLAST annotation summary of assembled *H*. *lupulus* transcripts.

	Transcripts annotated	
Database / BLAST algorithm	Number	Percentage
NCBI nt / BLASTn	62,342	41.44%
NCBI nr / BLASTx	43,645	29.01%
SWISS-Prot / BLASTx	33,492	22.26%
KOG / BLASTx	41,271	27.43%
All unique annotated sequences	82,482	54.83%

Further annotation and gene ontology classification was performed with Blast2GO analysis augmented by InterPro domain searches of *H*. *lupulus* transcripts. The comparison of hop transcripts against NCBI’s nr proteins revealed the top BLAST hit matches to *Prunus persica* (21.77%), *Vitis vinifera* (16.10%) and *Theobroma cacao* (9.61%), followed by other plant species (panel A in S1 Fig). The e-values for each sequence showed a peak at 10^−200^ and a distribution from 10^−5^ to 10^−30^ (panel B in S1 Fig). The hop transcript similarity values with database proteins were distributed from 45 to 100%, with a peak around 75%-85% (panel C in S1 Fig). The BLAST2GO annotation process is summarized in panel D in S1 Fig, with a total of 32,063 transcripts (21.31%), with 216,301 assigned GO term annotations. The majority of functional predictions (1,392,999) were obtained from the UniProt Knowledge Base (KB) (1,200,324) and the Arabidopsis Information Resource (TAIR) (126,085) (panel E in S1 Fig).

**Fig 1:**
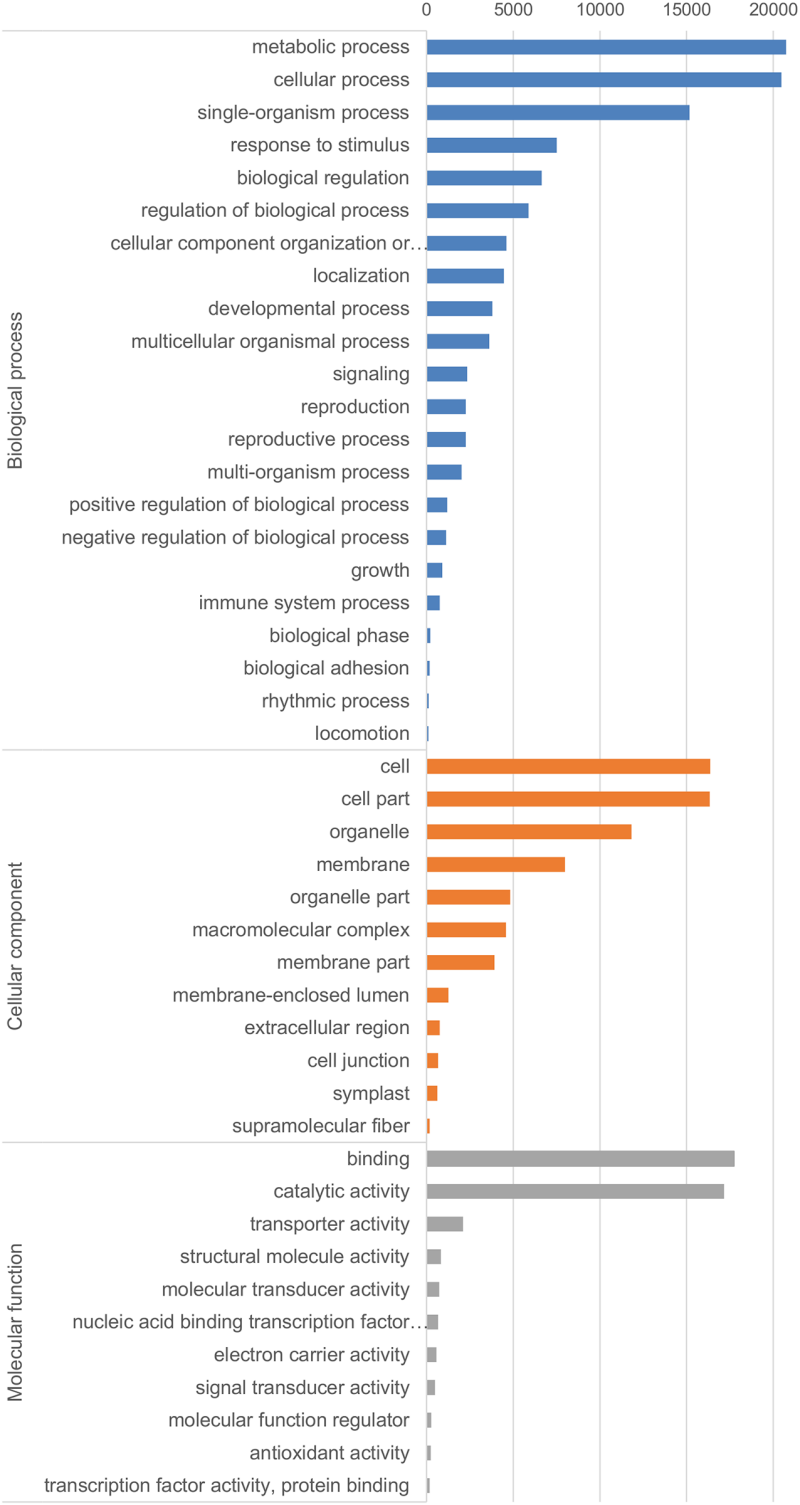
GO classification level 2 of *H*. *lupulus* annotated transcripts for three main GO categories: Biological process (BP), cellular component (CC) and molecular function (MF). Only classifications with > 100 sequences are shown.

The assigned GO terms were distributed into three GO classes: Biological Processes (128,942 terms, 59.61%), Cellular Function (33,418 terms, 15.45%) and Molecular Function (53,941 terms, 24.94%). Major sub-categories for GO level 2 classifications are presented in Fig 1. In Biological Processes, three GO level 2 categories represented 52.96% of all 27 assigned GOs: ‘metabolic process’ (GO:0008152, 19.48%), ‘cellular process’ (GO:0009987, 19.22%), and ‘single-organism process’ (GO:0044699, 14.25%). The class Cellular Component contained four major GO level 2 categories, amounting to 75.32% of 22 assigned GOs: ‘cell’ (GO:0005623, 23.47%), ‘cell part’ (GO:0044464, 23.44%), ‘organelle’ (GO:0043226, 16.96%) and ‘membrane’ (GO:0016020, 11.46%). Two main GO level 2 categories (85.09%) out of 17 were characteristic of the class Molecular Function: ‘binding’ (GO:0005488, 43.23%) and ‘catalytic activity’ (GO:0003824, 41.85%).

**Fig 2:**
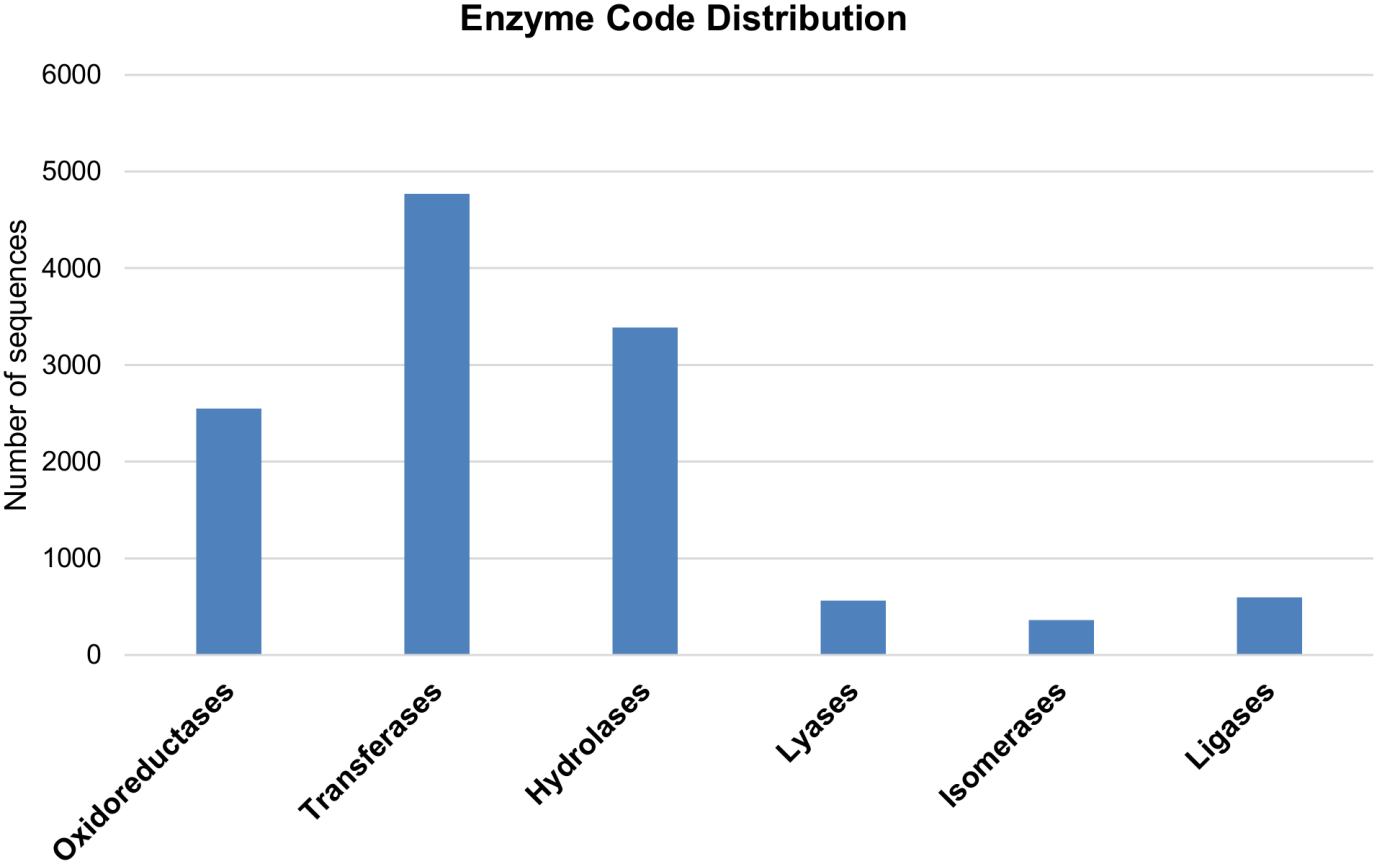
Top level enzyme codes assigned to 12,226 sequences.

Scheme numbers (ECs) could be assigned for 12,226 (38.13%) annotated sequences with GO term classification. The top level codes assigned to enzymes were distributed as follows: 38.99% to transferase activity, 27.69% to hydrolase activity, 20.86% to oxidoreductase activity, 4.87% to ligase activity, 4.60% to lyase activity and 2.97% to isomerase activity (Fig 2). InterPro search revealed that 27,714 hop transcripts had a hit to at least one InterPro database entry represented by domain (2479), family (2341), repeat (60) or site (403). An eukaryotic signal peptide cleavage site (signal-P) was predicted in 6,785 hop transcripts, while 6,287 transcripts were characterized by the presence of transmembrane helices.

### Target prediction and target selection of vd-sRNA

**Fig 3:**
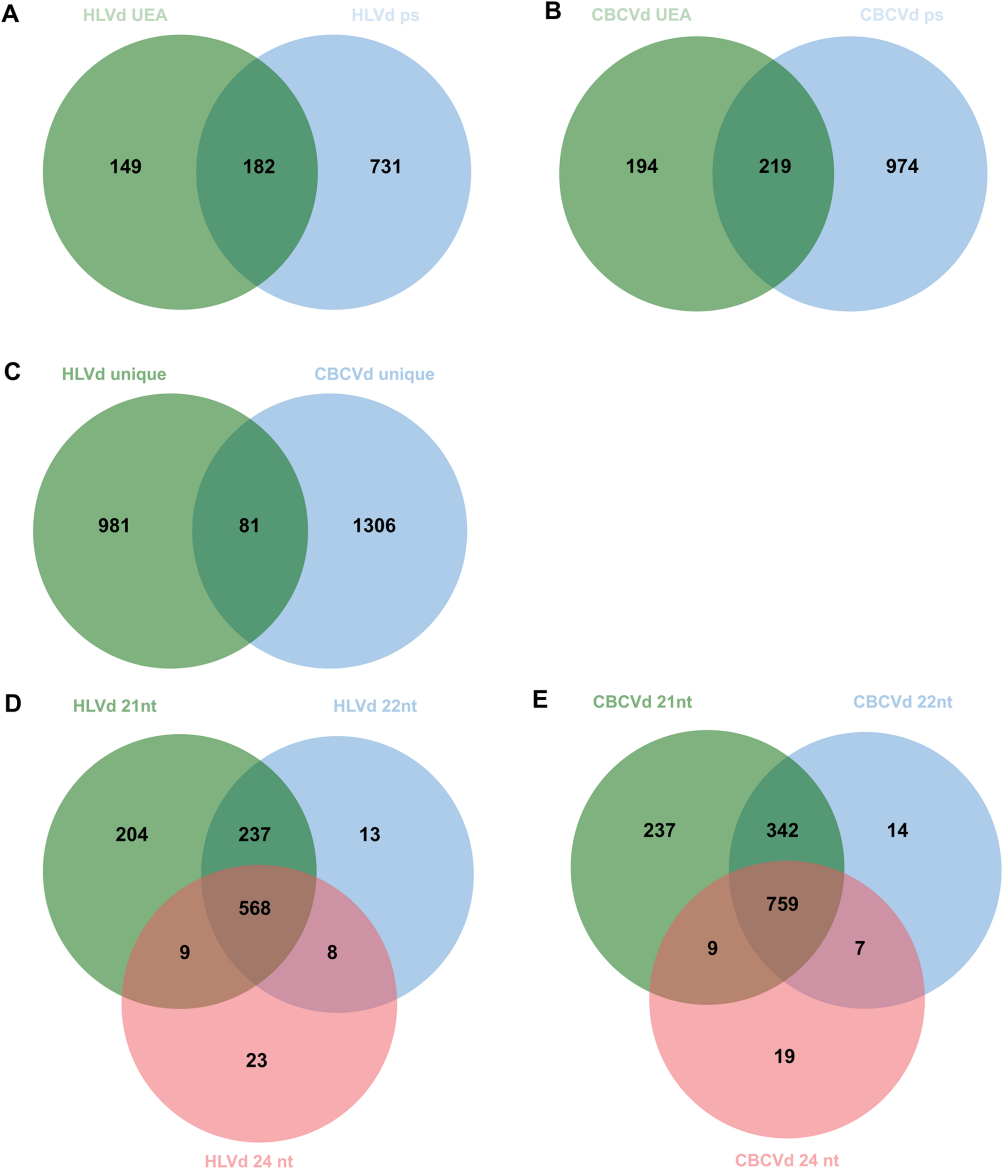
Results of target predictions for CBCVd and HLVd derived small RNAs. (A) Unique and shared targets for HLVd derived small RNAs predicted by UEA sRNA workbench (HLVd UEA) and psRNA Target (HLVd ps). (B) Unique and shared targets for CBCVd derived small RNAs predicted by UEA sRNA workbench (CBCVd UEA) and psRNA Target (CBCVd ps). (C) Only 81 of targets were in common predicted by two groups of vds-RNAs. (D) and (E) Shorter class of vd-sRNA for both viroids (21 nt, HLVd and CBCVd) revealed more targets than 22 and 24 nt class.

For hop target prediction of vd-small RNAs, we took into account possible lengths of HLVd- and CBCVd- derived sRNAs of 21, 22 and 24 bp long, the circular nature of viroid molecules and the presence of both orientations in the cell due to the (-) intermediate rolling circle amplification. In-silico cutting of the HLVd 256 nt sequence (GenBank X07397) yielded 512 possible 21, 22 or 24 nt sequences, while the CBCVd 284 nt sequence (GenBank KM211546) yielded 568 possible 21, 22 or 24 nt sequences. Two target prediction tools, UEA sRNA Workbench [38] and psRNA Target [39], predicted 1,062 and 1,387 unique targets for HLVd and CBCVd vd-sRNAs action. However, the tools significantly differed in the number of revealed hop targets, with psRNA Target predicting approximately three times more targets than the UEA sRNA tool, for both viroids, and approximately half of the targets predicted by the UEA sRNA tool were also revealed by the psRNA Target tool (Fig 3A and 3B). Between the two groups of vd-sRNAs only a small proportion of predicted targets were in common (81), revealing altogether 2,368 unique hop targets for possible sRNA action of these two studied viroids (Fig 3C). As expected, shorter vd-sRNAs (21 bp) revealed more targets than 22 and 24 bp ones, with a high proportion of mutual sharing (Fig 3D and 3E).

Comparison of Blast2GO annotation statistics of the set of predicted targets with the whole transcriptome revealed twice as high a proportion of predicted targets with B2GO parameters of annotation (S2 Fig). However, in a comparison of the most abundant proportions of the three GO level 2 classes of the whole transcriptome data with classes of predicted target data for HLVd and CBCVd vd-sRNAs action, only slight enrichment for some classes could be noted (S3 Fig), implying that a particular vd-sRNA may target some of these processes. Comparison of the proportion of assigned enzyme codes did not reveal any relevant increase in specific classes of enzyme codes for vd-sRNA predicted targets (S4 Fig).

Altogether, 17 hop targets were selected based on their annotation, with an emphasis on selecting genes of hormonal pathways (4), genes involved in small RNA biogenesis (2), genes targeted by CBCVd-derived sRNAs exhibiting high cellular concentrations (7) as revealed by viroid mapping data, and genes targeted by vd-sRNAs showing the highest level of complementarity with the target (4). Three targets were predicted as potential HLVd- and 14 sequences as potential targets of CBCVd-derived sRNA actions (Table 3, S1 Table). Five hop PR genes were additionally included (S2 Table).

**Table 3:** Seventeen selected hop transcripts as potential targets of vd-sRNA actions analysed by RT-qPCR.

No	Contig	Seq length	Viroid/vd-sRNA orientation	Prediction Tools	Transcript annotation	Group
1	30192	2042	HLVd / -	psRNATarget	phospholipase A1 (PLA1)	Metabolism of plant hormones
2	316	2396	HLVd / +	Both	linoleate 13S-lipoxygenase (LOX)	
3	51543	1395	CBCVd / -	psRNATarget	gibberellin 2-oxidase (GA2ox)	
4	24780	2290	CBCVd / +	psRNATarget	jasmonic acid-amino acid synthetase (JAR1)	
5	4643	5027	CBCVd / +	psRNATarget	endoribonuclease dicer homolog 3a (DCL3a)	Small RNA biogenesis
6	2112	9228	CBCVd /-,+	Both	endoribonuclease dicer homolog 1 (DCL1)	
7	29806	1124	CBCVd / +	psRNATarget	cold-regulated 413-plasma membrane protein 2 (COR413PM2)	CBCVd targets of vd-sRNAs with high cellular presence
8	44350	820	CBCVd / +	Both	GATA transcription factor (GATA)	
9	7166	2536	CBCVd / -	psRNATarget	acyl-CoA-binding domain-containing protein 5 (ACBD5)	
10	8499	1990	CBCVd / -	psRNATarget	uncharacterized protein 1 (UP1)	
11	11357	813	CBCVd / -	Both	multiprotein-bridging factor 1c (MBF1c)	
12	20309	4464	CBCVd / -, +	Both	putative polyprotein (Ppol)	
13	71362	215	CBCVd / -	Both	uncharacterized protein 2 (UP2)	
14	18517	7611	CBCVd / +	Both	uncharacterized protein 3 (UP3)	Genes with high complementarity with vd-sRNAs
15	21090	537	CBCVd / +	Both	AP-3 complex subunit sigma (AP3)	
16	24148	1065	CBCVd / +	Both	splicing factor 3B subunit 4 (SF3B)	
17	10708	1747	HLVd / +	Both	protein trichome birefringence-like 24 (TBL24)	

### RT-qPCR experiment on selected targets

PCR primers for the amplification of 17 selected hop transcripts were developed from annotated transcripts. In addition, 5 primers for expression analysis of hop PR genes were included [46] (S2 Table). Primers were designed to amplify a region of transcripts of an exact length of 90 bp, except for two transcripts (DCL1 62 bp and Ppol 67 bp), and to avoid annealing to regions of conserved protein domains. Optimal conditions for qPCR were defined during the optimization step as a 5 ng concentration of cDNA template and 300 nM concentrations of primers. For defined targets, successful amplification was achieved with a single dissociation curve and gel analysis confirming a single amplicon. All 17 successfully amplified targets and 5 hop PR genes showed no PCR inhibitions, with appropriate PCR efficiencies between 91.29% and 106.26%, as obtained from five-fold dilution series from bulked cDNA samples (S2 Table).

**Fig 4:**
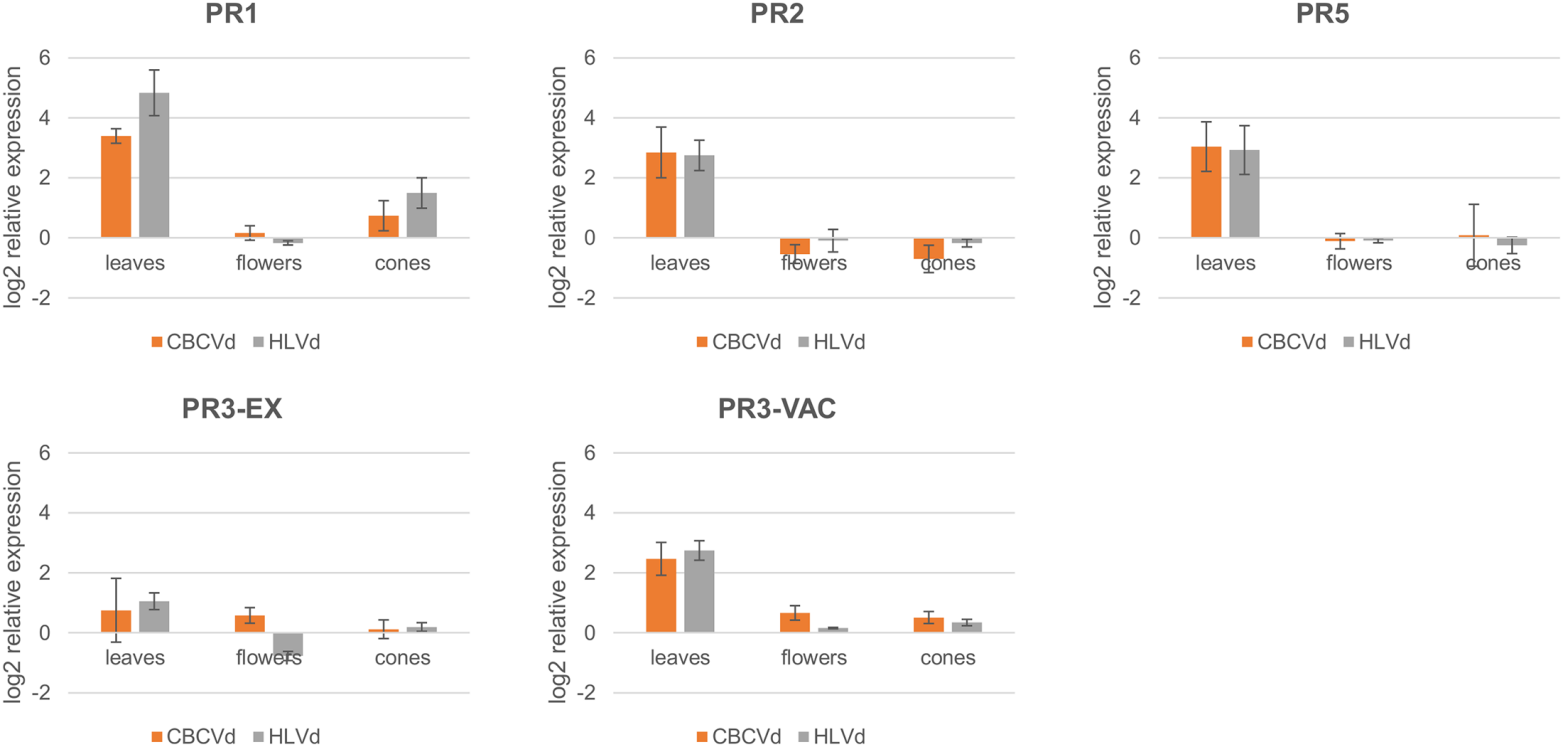
Relative expression levels (log_2_ scale) of five hop PR genes in leaves, flowers and cones in HLVd and CBCVd infected hop plants is strongly upregulated for 4 PR genes in leaves. The expression is relative to viroid-free plants. Error bars represent standard error of the mean (SEM) of biological replicates.

Expression analysis (FC>1.5) of PR genes (Fig 4) showed extremely high expression compared to viroid free plants for four out of five of these genes in leaves (PR1, PR2, PR3-VAC and PR5), being highest for the PR1 gene (HLVd: 28.6 and CBCVd: 10.5 FC), while for the other three genes, FC values were between 5.53 (PR3-VAC CBCVd) and 8.22 (PR5 CBCVd). The PR3-EX gene exhibited the smallest upregulation in leaves (HLVd: 2.08 and CBCVd: 1.68 FC), showing the smallest effect of viroid infection on expression of this particular gene. Interestingly, analysis of these genes in flower and cone tissues did not reveal strong up or down expression differences (>1.5 FC), except for PR1 and PR3-VAC, which were slightly upregulated in cones and flowers, respectively (HLVd: 2.82 and CBCVd: 1.67 FC for PR1 and CBCVd: 1.58 FC for PR3-VAC), while PR2 and PR3-EX were slightly downregulated in cones and flowers, respectively (CBCVd: 1.63 FC for PR2 and HLVd: 1.71 for PR3-EX). Tissue differences were highest for PR1 in the case of HLVd infection, whereby the difference in response between leaves and flowers and leaves and cones was 25.4 and 10.1 FC, respectively.

**Fig 5:**
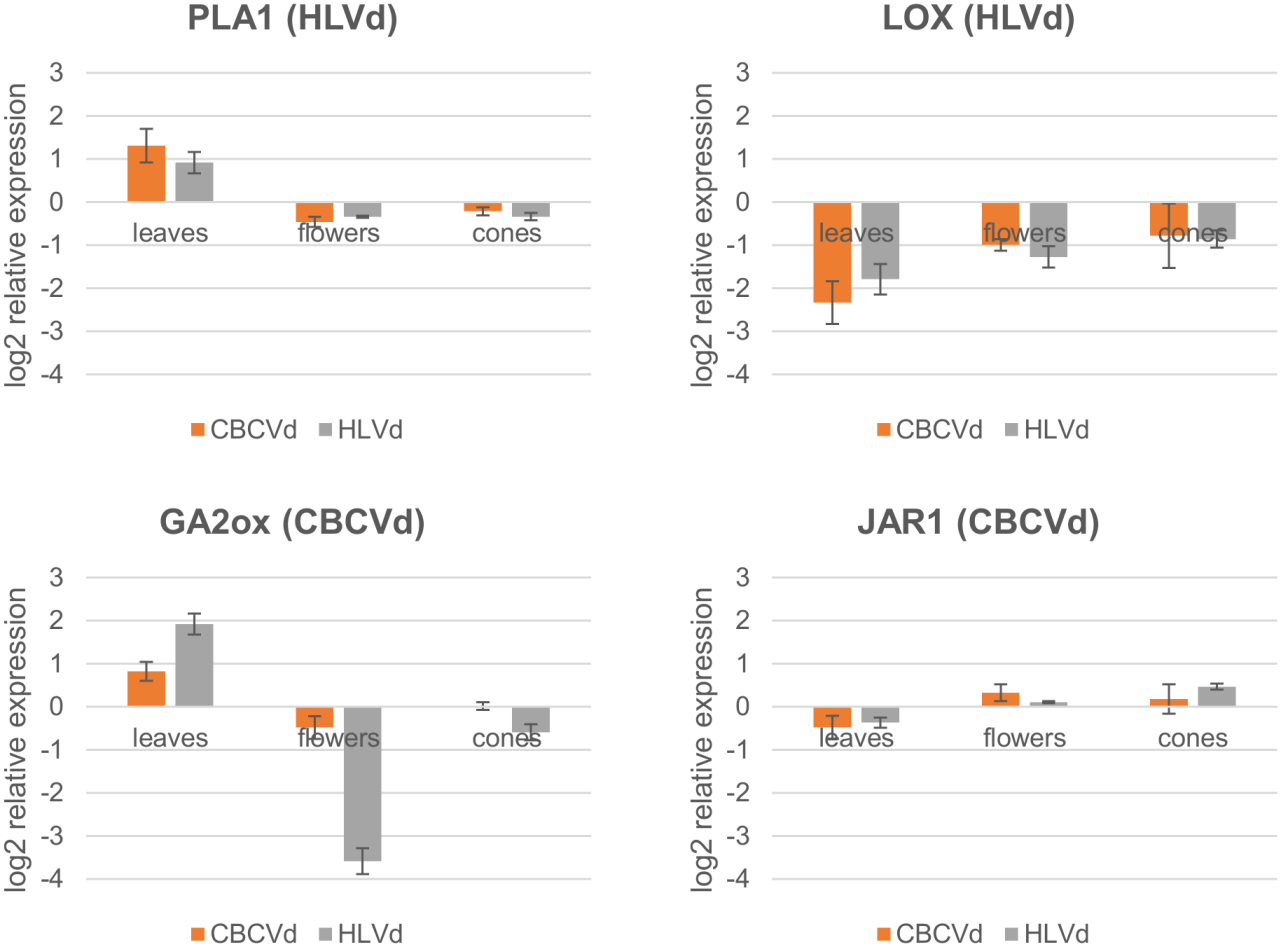
Relative expression levels (log_2_ scale) of four hop hormonal pathway transcripts in leaves, flowers and cones in HLVd and CBCVd infected hop plants. The expression is relative to viroid-free plants. Error bars represent standard error of the mean (SEM) of biological replicates.

Expression analysis was further performed for selected hormonal pathway targets with modelled evidence of interaction between the hop transcript and vd-sRNA. Downregulation of expression is expected in the case of confirmed silencing. These four targets, phospholipase A1 (PLA1), linoleate 13S-lipoxygenase (LOX), gibberellin 2-oxidase (GA2ox) and jasmonic acid-amino acid synthetase (JAR1) (Table 3, S1 Table, Fig 5) are involved in jasmonate and gibberellin hormonal pathways. Two of them are targeted by HLVd- and two by CBCVd-derived sRNAs (Table 3). LOX (HLVd targeted) showed a trend of downregulation in all three tissues for both viroid infected groups of plants, with the highest FC in leaves (HLVd: 3.47, CBCVd: 5.06), while flowers and cones exhibited FC in a range from 2.42 (HLVd flowers) to 1.72 (CBCVd cones). Downregulation in both viroid infected groups of plants may be due to HLVd derived sRNA action, since CBCVd plants are also HLVd infected. Expression of the next transcript PLA1 (HLVd targeted) was characterized by upregulation in leaves for both groups of viroid infected plants (HLVd: 1.88 and CBCVd: 2.47 FC), with a minimum effect of downregulation in flowers and cones (<1.5 FC). JAR1 (CBCVd targeted) showed minimum downregulation in leaves and minimum upregulation in flowers and cones (<1.5 FC) in analysed tissues and groups of viroid infected plants. GA2ox (CBCVd targeted) was upregulated in leaves for both viroid infected groups of plants with a higher trend in HLVd infected plants (HLVd: 3,78 and CBCVd: 1,76 FC). Interestingly, although the transcript was predicted as a CBCVd sRNA target, it showed very strong downregulation in flowers of HLVd infected plants (12.05 FC), and a much smaller one in cones of the same plants (1.51 FC). In cones of CBCVd infected plants, expression analysis did not reveal a difference from viroid free plants, while it showed a trend of downregulation in flowers (<1.5 FC).

**Fig 6:**
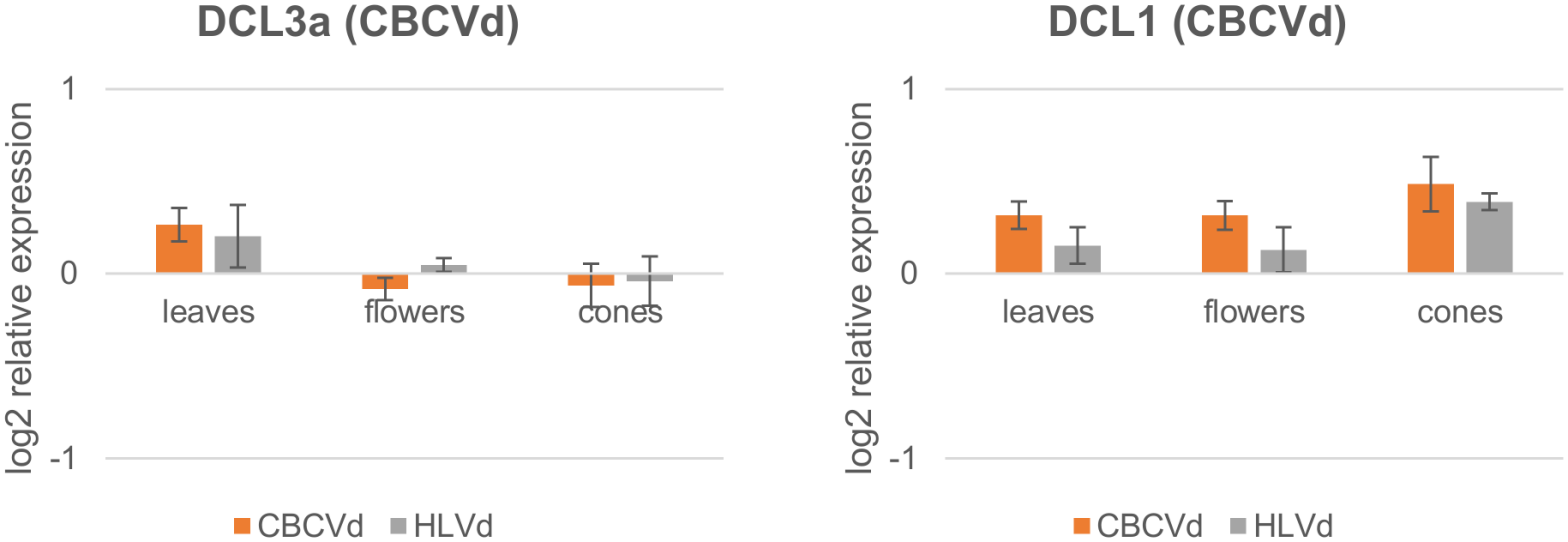
Relative expression levels (log_2_ scale) of two hop DCL transcripts involved in RNAi biogenesis in leaves, flowers and cones in HLVd and CBCVd infected hop plants displays stable expression profiles. The expression is relative to viroid-free plants. Error bars represent standard error of the mean (SEM) of biological replicates.

Two transcripts with *in-silico* evidence of interaction with CBCVd derived sRNA belonged to hop RNAi biogenesis machinery (Table 3, S1 Table, Fig 6): endoribonuclease dicer homolog 3a (DCL3a) and endoribonuclease dicer homolog 1 (DCL1). Interestingly, both genes showed very stable expression, with minimum expression fluctuations in different tissues and in the two groups of viroid infected plants (<1.5 FC).

**Fig 7:**
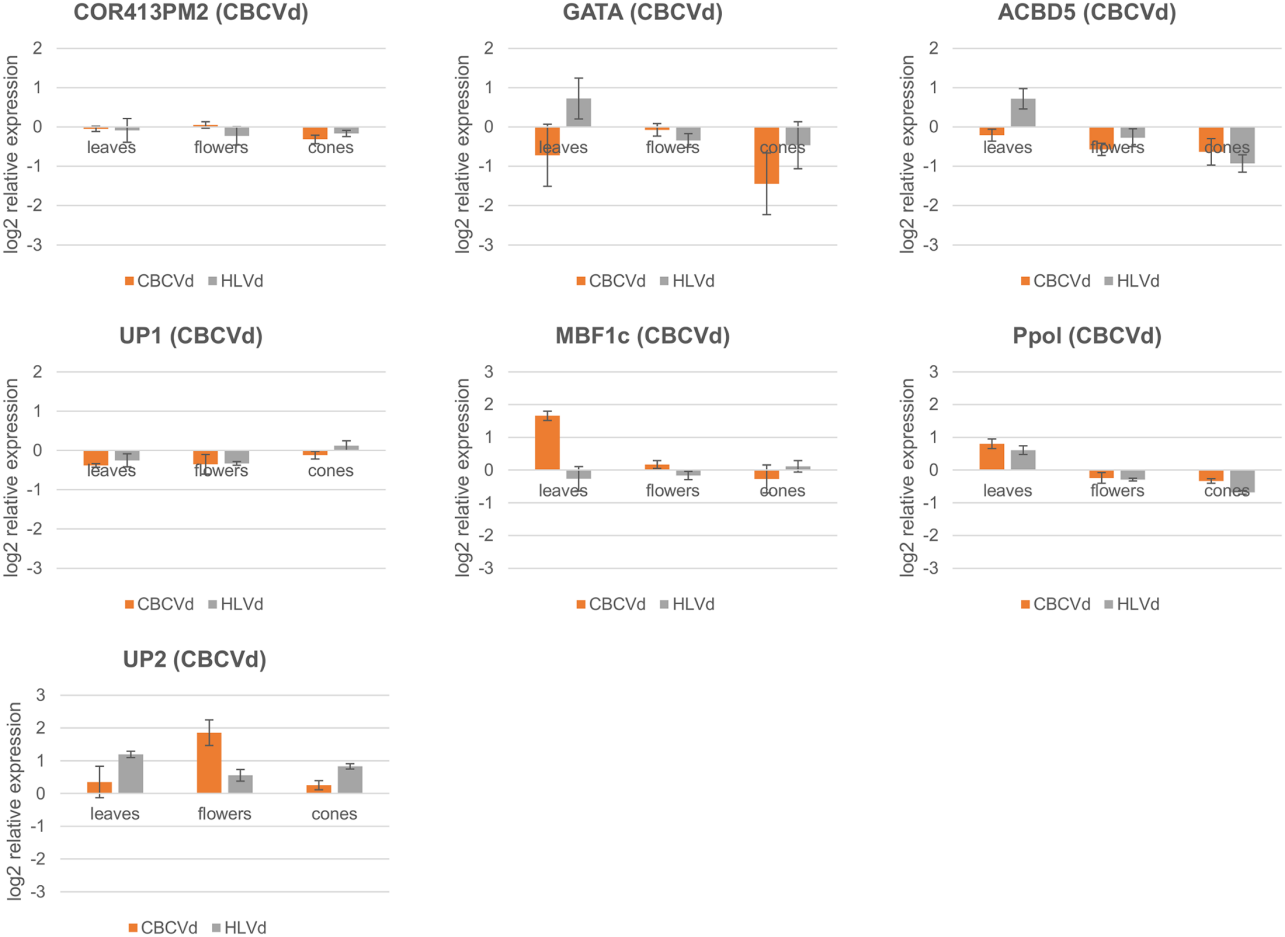
Relative expression levels (log2 scale) of seven hop transcripts selected based on high cellular presence evidence of CBCVd derived sRNAs in HLVd and CBCVd infected hop plants in leaves, flowers and cones. Expression profile of GATA transcription factor for CBCVd infected plants is showing possible vd-sRNA action of downregulation. The expression is relative to viroid-free plants. Error bars represent standard error of the mean (SEM) of biological replicates.

The next group of seven targets, cold-regulated 413-plasma membrane protein 2 (COR413PM2), GATA transcription factor (GATA), acyl-CoA-binding domain-containing protein 5 (ACBD5), multiprotein-bridging factor 1c (MBF1c), putative polyprotein (Ppol) and two uncharacterized proteins (UP1 and UP2) (Table 3, S1 Table, Fig 7) were selected based on CBCVd-derived sRNAs, which were characterized by high cellular presence in infected hop plants, as revealed by mapping profiles [14]. For this group of targets, one of them—GATA transcription factor (GATA)—exhibited an effect of downregulation by CBCVd derived sRNAs in all three tissues. It was downregulated in leaves of CBCVd infected plants (1.65 FC), while upregulated in leaves of HLVd infected plants (1.65 FC). The effect of downregulation was seen in cones of both groups of plants, being stronger in CBCVd (>1.5) infected plants (HLVd: 1.38 and CBCVd: 2.73 FC), while in flowers, downregulation was almost negligible (<1.5) (HLVd: 1.27 and CBCVd: 1.05 FC). ACBD5 showed small downregulation in leaves of CBCVd infected plants (<1.5) (1.16 FC) but was upregulated in leaves of HLVd infected plants (1.65 FC), while both group of plants showed stronger downregulation in flowers and cones, being higher in cones of HLVd infected plants (HLVd: 1.90 and CBCVd: 1,55 FC). COR413PM2 showed either slightly increased or decreased expression levels in all tissues compared to viroid free plants (<1.5 FC). A similar trend was seen for UP1, with slight downregulation of transcript in leaves and flowers in both groups of plants (up to 1.30 FC in CBCVd) and minimum change in cones. Interestingly, in contrast to our hypothesis of downregulation of targets by vd-sRNA, transcript MBF1c was highly expressed in leaves of CBCVd infected plants (3.16 FC), while slightly downregulated in HLVd infected plants (1.20 FC) and with minimum expressed fluctuations in flowers and cones. Ppol and UP2 showed the same expression trend in tissues of both groups of viroid infected plants. While the first target had higher expression in leaves (HLVd: 1.52 and CBCVd: 1.74 FC), it was slightly downregulated in flowers and cones, and the second target showed higher expression in all tissues compared to viroid free plants, with the highest expression in flowers of CBCVd infected plants (3.62 FC) and in leaves and cones of HLVd infected plants (HLVd leaves: 2.28 and HLVd cones: 1.77 FC).

**Fig 8:**
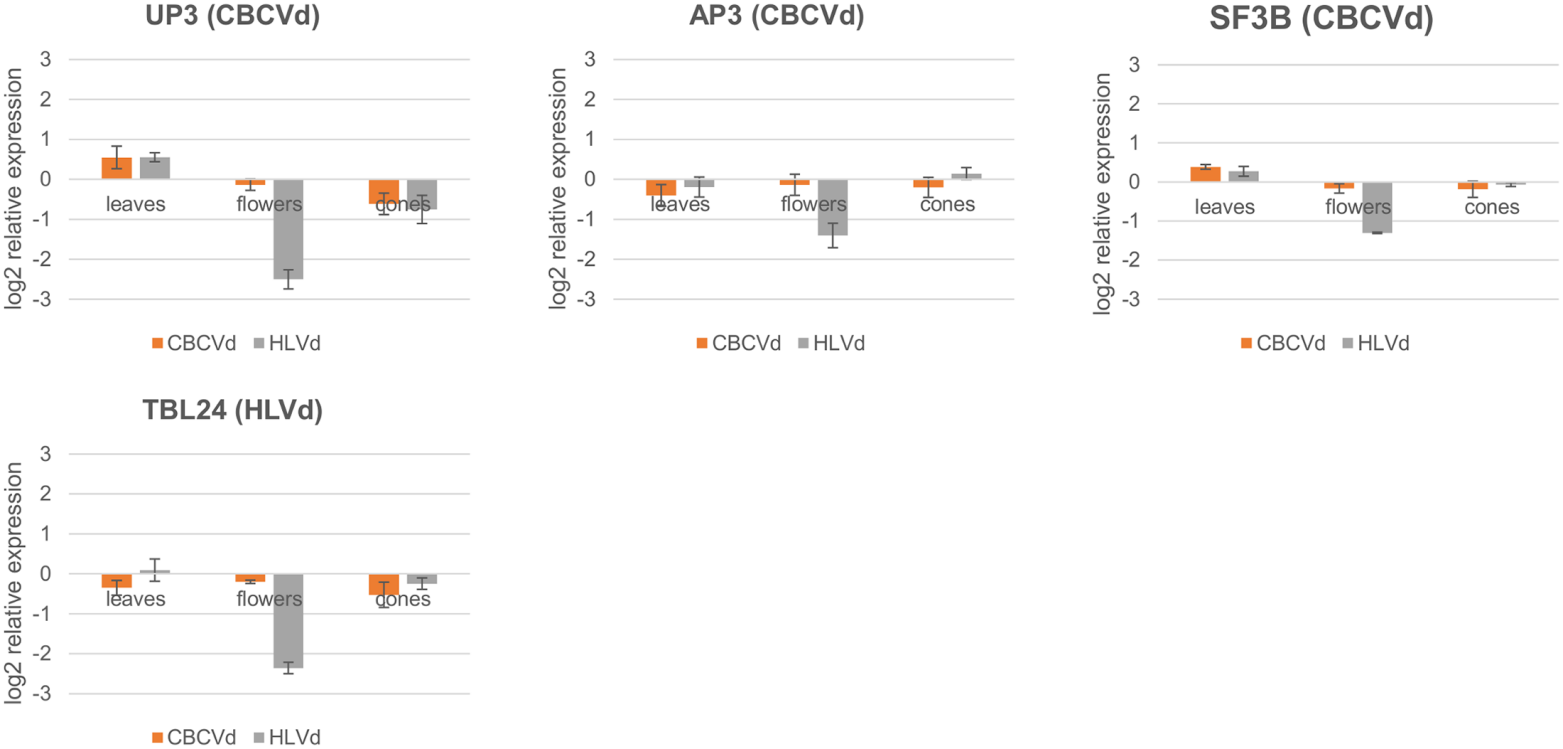
Relative expression levels (log2 scale) of four hop transcripts showing the lowest expectation scores of three CBCVd and one HLVd derived sRNAs in infected hop plants in leaves, flowers and cones in HLVd and CBCVd infected hop plants. The expression is relative to viroid-free plants. Error bars represent standard error of the mean (SEM) of biological replicates.

The last four targets were selected on the basis of the lowest computed expected scores provided by the psRNA target algorithm [39] between hop transcripts and vd-sRNAs uncharacterized protein 3 (UP3), AP-3 complex subunit sigma (AP3), splicing factor 3B subunit 4 (SF3B) and protein trichome birefringence-like 24 (TBL24) (Table 3, S1 Table, Fig 8). All three CBCVd derived sRNA targets exhibited unexpectedly high downregulation in flowers of HLVd infected plants (5.67, 2.65 and 2.47 FC), while the expected downregulation effect in flowers of CBCVd infected plants was negligible (FC<1.5) (1.10, 1.10 and 1.12 FC). A similar effect was also characteristic for HLVd derived sRNA target TBL24, in which downregulation in flowers of HLVd infected plants showed 5.13 FC compared to viroid free plants. Both up- and downregulation was observed for these targets in leaves and cones. The first transcript, uncharacterized protein UP4, was upregulated in the leaves of both groups of viroid infected plants, with comparable fold changes (HLVd: 1.47 and CBCVd: 1.46 FC) and similarly downregulated in cones. The second CBCVd derived sRNA target, AP3, was slightly downregulated in the leaves of both groups of viroid infected plants (HLVd: 1.15 and CBCVd: 1.32 FC) and showed minimum up- and downregulation in cones (HLVd upregulation: 1.15 and CBCVd downregulation: 1.32 FC). SF3B, also a predicted target of CBCVd derived sRNA action, was slightly upregulated (<1.5) in the leaves of both groups of viroid infected plants (HLVd: 1.21 and CBCVd: 1.31 FC) and showed negligible downregulation in cones (HLVd: 1.05 and CBCVd: 1.14 FC). The target of HLVd derived sRNA, TBL24, had comparable expression in leaves of HLVd infected plants (1.07 FC) and was slightly downregulated in CBCVd infected plants (1.28 FC), while it was downregulated in cones, with a stronger effect in CBCVd infected plants (HLVd: 1.19 and CBCVd: 1.44 FC).

## Discussion

We *de-novo* assembled an RNA-seq derived hop transcriptome, defined targets of HLVd and CBCVd derived sRNAs and selected a panel of potential targets together with PR genes, whose expression was monitored using RT-qPCR. This allowed us to compare the hop response to infection by the aforementioned two viroids. Indeed, *in-silico* target prediction showed that possible targets exist in the hop transcriptome for vd-sRNAs and the majority of selected targets showed differential expression in viroid infected plants compared to viroid-free plants, suggesting transcriptome changes due to the infection.

To elucidate possible pathogenicity mechanisms of aggressive CBCVd and symptomless HLVd infections of hop plants, we started with computational predictions of vd-sRNAs targeted genes of hop. For this purpose, we developed a detailed hop plant NGS transcriptome represented by 150,443 transcripts expressed in different hop tissues from several developmental stages. Hop transcriptomes were already assembled and studied in the past by Sanger chemistry, but mainly focused on glandular trichomes to study lupulin specific transcripts [48,49]. A similar study employing an NGS Illumina RNA-seq approach further expanded knowledge about lupulin gland-specific biosynthesis of precursors for the production of bitter acids. In that study, a transcriptome from leaves, cones and lupulin glands was assembled and ~ 170,000 contigs were obtained, which is a similar number to our study. The authors reported 745 bp to be the average length of the transcripts, which is better than was achieved in our study (494 bp) [50]. The number of hop assembled transcripts is fairly high compared to some Illumina transcriptome *de-novo* assembly studies of other crops, such as peanuts (59,077) [51], celery (42,280) [52] or carrots (58,751) [53]. The higher number of assembled transcripts probably results from the highly heterozygous nature of outcrossing hops, which contributed to separate assemblies of some allelic variants, the presence of alternative spliced transcripts, and transcripts being assembled into several split contigs. The recently available unannotated draft assembly of the hop genome will certainly improve future efforts in hop transcript reconstruction [54]. Further BLAST analysis against the available nucleotide and protein databases showed that 41.4%, 29%, 22.3% and 27.4% of hop transcripts have similar sequences in NCBI nt, NCBI nr, SWISS-Prot and KOG databases, respectively. If transcripts shorter than 200 bp were not taken into account, the numbers improved, especially for protein databases (nr 39.0%, SWISS-Prot 30.2%, KOG 36.9%). Similar observations about shorter contigs lacking hits in protein databases have also been made in other *de-novo* assembly transcriptome studies [55]. Transcripts without revealed similarities to known proteins probably belong to noncoding RNA species, 5’ or 3’ untranslated regions, short sequences with non-significant protein hits or are simply assembly artefacts. BUSCO analysis [35] of the transcriptome achieved 89% and confirmed its near completeness. This software, intended to assess transcriptome assembly and annotation completeness by comparison of transcripts to selected Universal Single-Copy Orthologs expected to be always present in all cells, is becoming a popular tool for NGS generated transcriptomes [56]. Functional annotation assigned different GO categories to the hop transcripts (Fig 1). These data are valuable for researchers interested in specific pathways or in transcripts controlling traits of interest. This part of the research clearly showed that NGS sequencing is an efficient strategy for transcriptome reconstruction of a highly heterozygous non-model organism such as hop. The sequences generated in this study can be used for additional studies, such as SNP or microsatellite marker development, mapping studies or in hop breeding programs aided by marker assisted selection tools.

In the next step, we performed *in-silico* target prediction for HLVd and CBCVd derived sRNAs. There are many small/micro RNA target prediction computational tools available (reviewed in [57]) and some of them have also been extensively and systematically evaluated [58]. They focus on different parameters of the target recognition process, which are usually plant or animal specific, and methods of discovery, which are either similarity based or based on machine learning approaches. Two different software packages were used, ps-RNA target [39] and UEA sRNA workbench [38], both of them classified as plant specific tools. However, they predicted widely different numbers of targets (Fig 3A and 3B). Comparison of various small RNA target predication tools has already highlighted different efficiencies and sensitivities for the tested tools. Authors have also indicated the diversity of newly discovered and non-conventional small RNA-mRNA interaction features, which can also be species specific and not included in predicting algorithms [58]. While UEA sRNA workbench did not provide any selection parameters, some are available for the ps-RNA target tool, which can affect the number of predictions. We used the default parameters for the ps-RNA target tool. When the ‘maximum expectation’ value was lowered to more stringent prediction values, set at 2, 1 and 0, significantly fewer numbers were predicted.

We selected the qRT-PCR method for monitoring expression of selected hop transcripts (Table 3, S1 Table) in three tissues (leaves, flowers and cones), since it is the method of choice for precise measurement of a smaller number of targets [59].

Expression of PR-genes, which are known to be part of the general host defence response activated against pathogens, including bacteria, fungi and viruses [60], has been investigated in hop-viroid interactions. Four hop PR genes (Fig 4) showed strong accumulation of PR transcripts for both groups of viroid infected plants in leaves, while their activation was not triggered in flowers or cones, except for gene PR-1 activation in cones. Tissue and developmental specific expression of *A*. *thaliana* PR-1 proteins were confirmed in a microarray experiment of healthy plants [60], which might also be the cause of the lower expression that we encountered in flowers and hop cones. It has already been shown in several tomato-viroid studies that plants infected by PSTVd or CEVd have elevated expression of several PR proteins [61,62]. In our case, even symptomless HLVd hop plants showed elevated expression of PR proteins. Although HLVd does not cause visible symptoms, it presumably interferes with secondary metabolite production of lupulin glands [63], and its cellular replication probably activates a PR protein response. Interestingly, it was recently suggested by RNA-seq analysis that sensitivity to HSVd in hops is related to a PR protein downregulation expression profile, in a study of affected and tolerant hop cultivars [64], a phenomenon not observed in our case.

Traditionally, based on pioneering articles on the discovery of HLVd by Puchta et al. [4] or on HLVd testing by Barbara et al. [5], it is widely assumed that hop cones are the most infected organs in the case of HLVd, since cone tissue was used for RNA isolation in most samples. However, no research has to date been published in terms of the concentration distribution of hop viroids in different parts of hop plants or organs using qPCR measurements. Small RNA NGS sequencing of bulked RNA tissue of plants infected by both viroids by our research group confirmed extremely high and comparable cellular concentrations of vd-sRNAs for both viroids, totaling up to 3% of all reads, although this does not answer the question of their spatial distribution [14]. Research papers studying the distribution of viroids in other plant-viroid pathosystems have in fact shown evidence of viroids’ uneven distribution in the plant; for example, for peach latent mosaic viroid (PLMVd) [65]. Since both viroids, HLVd and CBCVd, in hops can easily be detected throughout the growing season in different sampled tissues, we believe that their concentration is comparable between different tissues. Our assumption was therefore that for transcripts targeted by vd-sRNAs, downregulation should be observed in all analyzed tissues.

It is well known that viral pathogens produce suppressors of the RNA silencing pathway in order to inhibit or target proteasome mediated proteins, important for degradation, of this antiviral part of the plant immune system [66,67]. For example, a plant’s DCL proteins can be inhibited by P38 or P1 suppressors of *Turnip crinkle virus* [68] and *Rice yellow mottle virus* [69]. The target prediction step indicated that two CBCVd-derived sRNAs may targets hop’s DCL3a and DCL1 transcripts (Table 3, S1 Table). However, expression analysis showed very stable and unchanged expression of these two genes in all analysed organs, demonstrating uncompromised activity of these two DCLs in infected plants (Fig 6). Therefore vd-sRNAs do not compromise the activity of these two important genes of the RNA silencing pathway. Their very stable expression suggests their possible use as biotic stress reference genes in hop RT-qPCR experiments [45].

Due to the sessile lifestyle of plants, their defence strategies against pathogens also depend on fine-tuned hormonal pathways, including three pillars of stress hormones (salicylic acid, jasmonates and ethylene), as well as growth hormones such as cytokinins, gibberellins and brassinosteroids. On the other hand, pathogens have evolved numerous virulence strategies targeting the accumulation of hormones or their signalling [70]. We therefore hypothesize that vd-sRNAs may target and silence any of the genes involved in the aforementioned hormonal pathways. It is already known from a microarray experiment on PSTVd infected tomato plants, that viroid infection induces complex changes affecting hormone signalling [71]. Complementarity between vd-sRNAs and 3 genes involved in the jasmonate pathway (PLA1, LOX and JAR1), and GA2ox involved in gibberellin production, was predicted and their expression was monitored by RT-qPCR (Fig 5). The first gene, PLA1, targeted by HLVd derived sRNA, had elevated expression in the leaves of both groups of viroid infected plants, confirming that a plant defence response was triggered but not silenced. However, expression of this gene was not impaired in flowers or cones, probably due to their specialized biological function. A growing body of evidence indicates that phospholipase A, as well as other phospholipid hydrolyzing enzymes, plays an important role in control of the plant defence response to attack by invading pathogens [72]. The second gene, LOX, targeted by HLVd derived sRNA, was downregulated in both groups and all tissues of viroid infected plants, with the highest effect in leaves (Fig 5). Downregulation of this particular enzyme was also confirmed in a tomato transgenic line expressing PSTVd derived sRNAs in the absence of viroid replication. Moreover, the expression change was similar to the change we detected in hop leaves (log2 expression change tomato -1.96, HLVd hop -1,80 and CBCVd hop -2,34) [71]. Our observation for this gene may be due to HLVd-derived sRNA targeting, since both groups of hop plants were HLVd infected. The third gene of the JA pathway, jasmonic acid-amido synthethase JAR1, targeted by CBCVd derived sRNA, showed minor fluctuations of expression compared to viroid free plants and seems not to be influenced by the disease status of the plant. Mutants with a defective JAR1 gene are of special interest and are important experimental models due to the reduced sensitivity to JA in studies of plant resistance [73]. The last gene, GA2-oxidase, was predicted to be targeted by CBCVd-derived sRNAs. However, expression profiles were different among tissues and viroid infected plants and did not show the expected trend of downregulation in all tissues (Fig 5). GA2-oxidase is known to be a dominant gibberellin catabolism gene and its overexpression contributes to dwarf genotypes in many plants, such as poplar [74] or *Solanum* [75] species. Dwarfism of hop plants is particularly exhibited in CBCVd infection, while plants are of normal height in the case of HLVd viroid infection. Upregulated expression of this gene in leaves in both groups of plants is probably not a major contributor to the dwarfism of the CBCVd infected plants.

It is known that siRNAs require higher complementarity with the target than do miRNAs [76]. We therefore selected the vd-RNAs (3 CBCVd and 1 HLVd derived) that were most identical to the targets and monitored their expression. The targets were annotated as uncharacterized protein UP3, AP3 (involved in intracellular protein transport and vesicle-mediated transport), SF3B (involved in mRNA splicing) and TBL24 (involved in cell wall organization or biogenesis) (Table 3, S1 Table). Unexpectedly, all four targets, even the three predicted as being targeted by CBCVd-derived sRNAs, were particularly downregulated in flowers of HLVd infected plants (Fig 8). Other biological processes than silencing by vd-sRNAs probably interfere with the downregulation of these targets in flower tissue of HLVd infected plants.

It is well documented that vd-sRNAs exist in hosts in varying numbers and those with higher prevalence come from defined hot-spot regions of the viroid’s genome [14]. In *in-vitro* studies of siRNA silencing, it was shown that there is a concentration-dependent gene silencing effect, with a higher concentration of sRNAs more successfully decreasing gene activity [77]. Seven hop targets were therefore chosen, based on CBCVd-derived sRNAs with supported evidence of having a high cellular content *in-planta* [14]. These hop transcripts are presumably involved in important processes, such as response to cold stress (COR413PM2) [78], regulation of transcription (GATA) [79], tolerance to heat and osmotic stress by partially activating the ethylene-response signal transduction pathway (MBF1c) [80,81], while two others are either poorly annotated (uncharacterized protein UP2) or show a probable retrotransposon origin (Ppol) (Table 3, S1 Table). Interestingly, we found evidence of possible downregulation of the GATA transcription factor (GATA) in this group of transcripts, in CBCVd infected plants showing decreased expression in leaves (1.65 FC) and in cones (2.73 FC), while in HLVd infected plants, this phenomenon was, as expected, not observed (Fig 7). A similar level of expression suppression of target mRNAs by PSTVd derived sRNAs was observed in tomato plants, in which callose synthase gene expression was suppressed 1.5-fold [24]. Suppression of expression of the transcription factor regulating an important biological process—the rate of transcription of genetic information—could probably manifest in severe disease symptoms, which are observed in the case of CBCVd infected hop plants.

The data presented in this paper demonstrate that NGS technology is an appropriate method of choice for quickly acquiring transcriptomes for non-model organisms such as hop. Furthermore, an in-silico approach revealed the existence of a high level of sequence similarity of vd-sRNAs of CBCVd causing severe stunt disease and non-harmful HLVd with hop transcripts. Elevated expression of PR proteins confirmed the disease status of both viroid infected groups of hops, even in the case of non-symptomatic HLVd. Expression analysis of targets selected based on several criteria showed altered expressions in different hop tissues, demonstrating that viroid infection can re-program the transcriptomic plan of healthy plants. Moreover, for two transcripts, we found evidence of possible downregulation of transcripts by HLVd and CBCVd derived sRNAs. This evidence will be further confirmed by an RLM-RACE or degradome experiment. However, a better understanding of vd-sRNA action is needed, together with defined methodologies on selection criteria for their action, for precise selection of the targets. We also have to take into account the possibility of other actions of vd-sRNAs, which might be different to the PTGS model. The results acquired in this research will pave new research paths in strategies to fight viroid diseases in hop production.

## Supporting information

S1 Fig Summary of Blast2GO annotation process. (TIFF)

S2 Fig Comparison of BLAST2GO results between the whole transcriptome, CBCVd and HLVd targets CBCVd and HLVd targets revealed better annotation statistics for contigs targeted by vd-sRNAs. (TIFF)

S3 Fig Comparison of GO classification level 2 of three GO categories for whole transcriptome, CBCVd and HLVd targets. (TIFF)

S4 Fig Comparison of shares of specific classes of enzyme codes for vd-sRNA predicted targets with whole transcriptome. (TIFF)

S1 Table Additional information on seventeen selected hop transcripts. (XLSX)

S2 Table Developed primer pairs and PR primers used in RT-qPCR analysis and their PCR efficiencies. (DOCX)
