# Supplementary figures and images for: Development of hop transcriptome to support research into host-viroid interactions
Source: PLoS One. 2017 Sep 8;12(9):e0184528. doi: 10.1371/journal.pone.0184528 (PMC5590963; doi:10.1371/journal.pone.0184528)

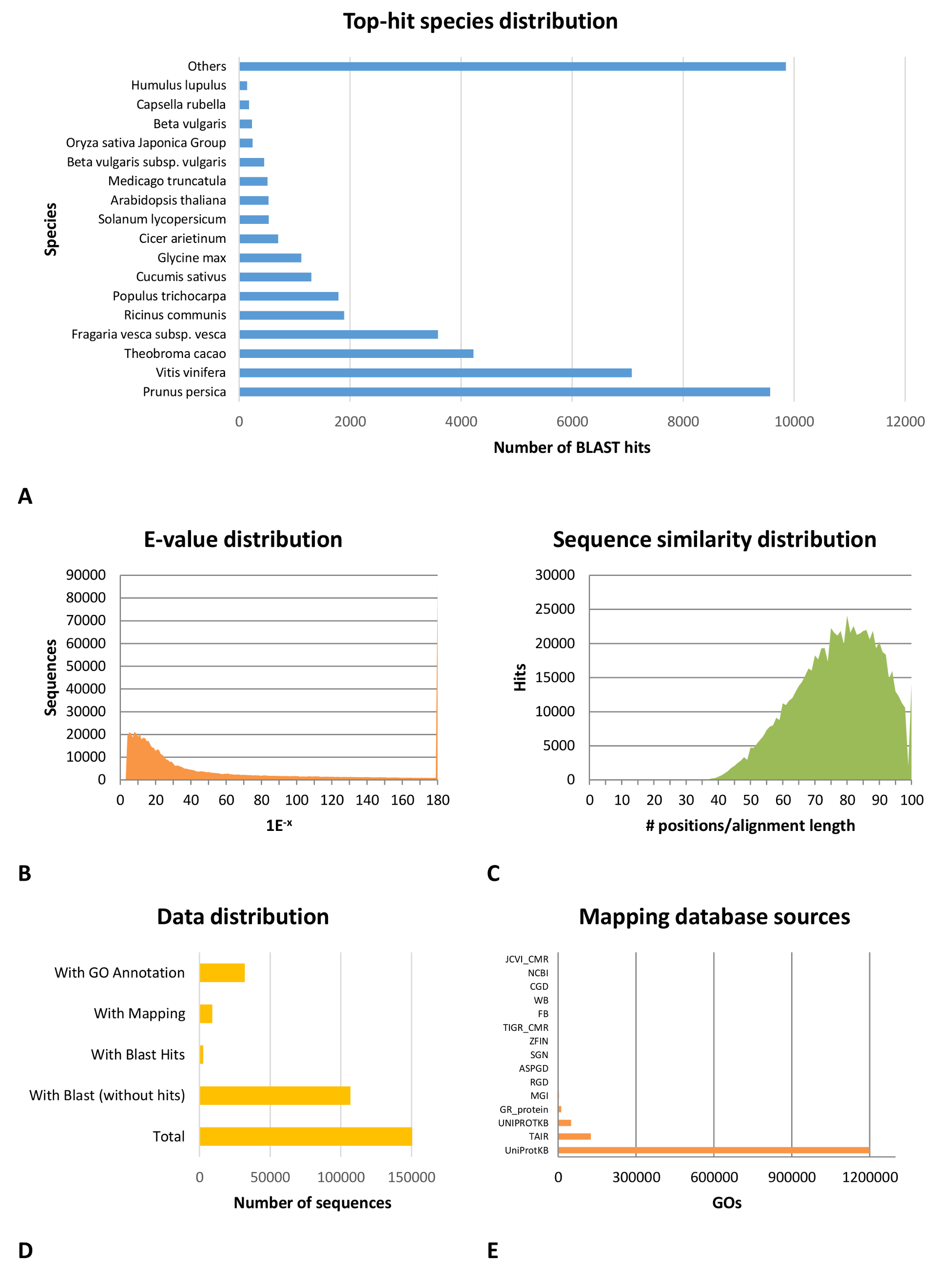

Supplement: S1 Fig — (TIFF) [file pone.0184528.s001.tiff]

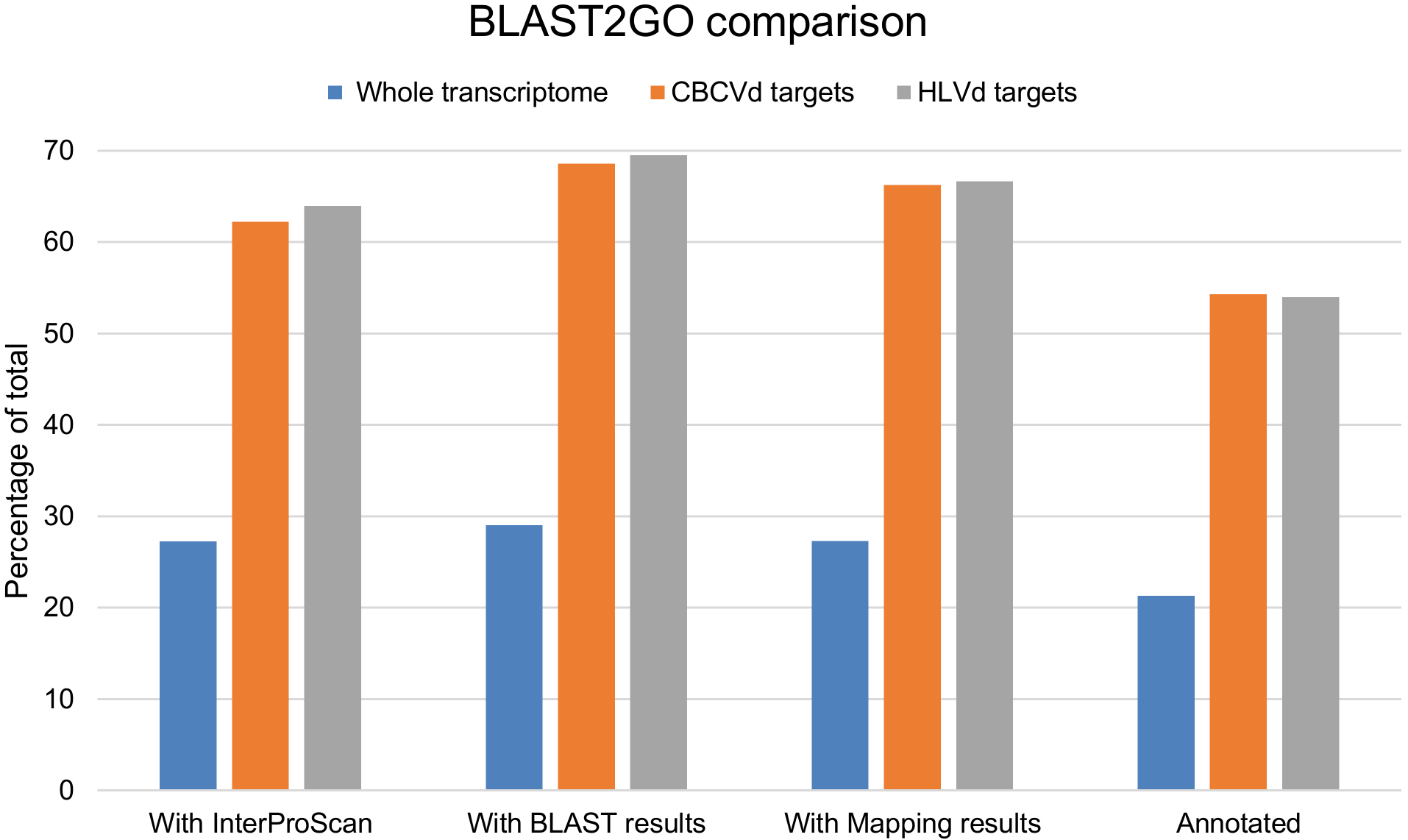

Supplement: S2 Fig — (TIFF) [file pone.0184528.s002.tiff]

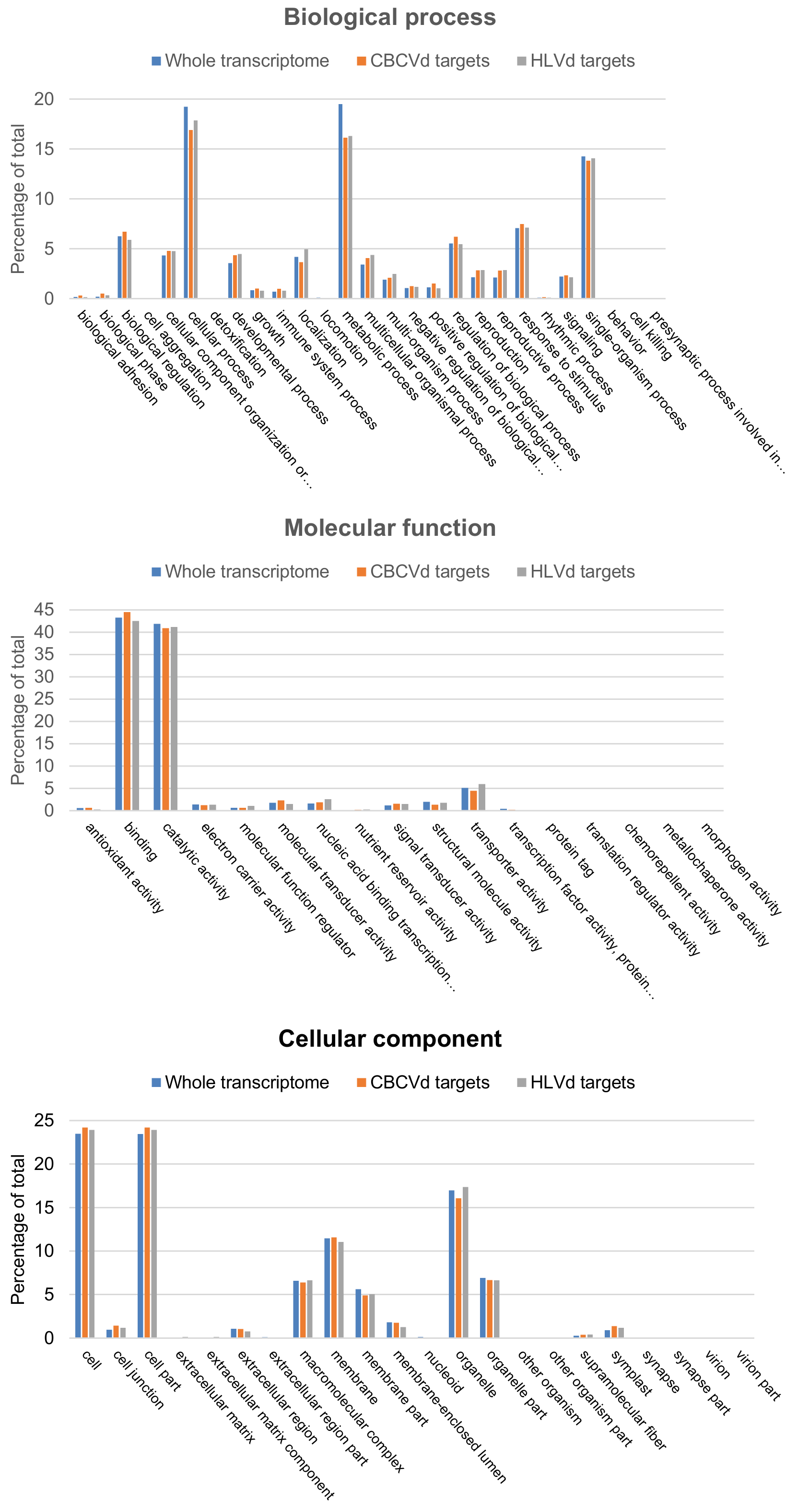

Supplement: S3 Fig — (TIFF) [file pone.0184528.s003.tiff]

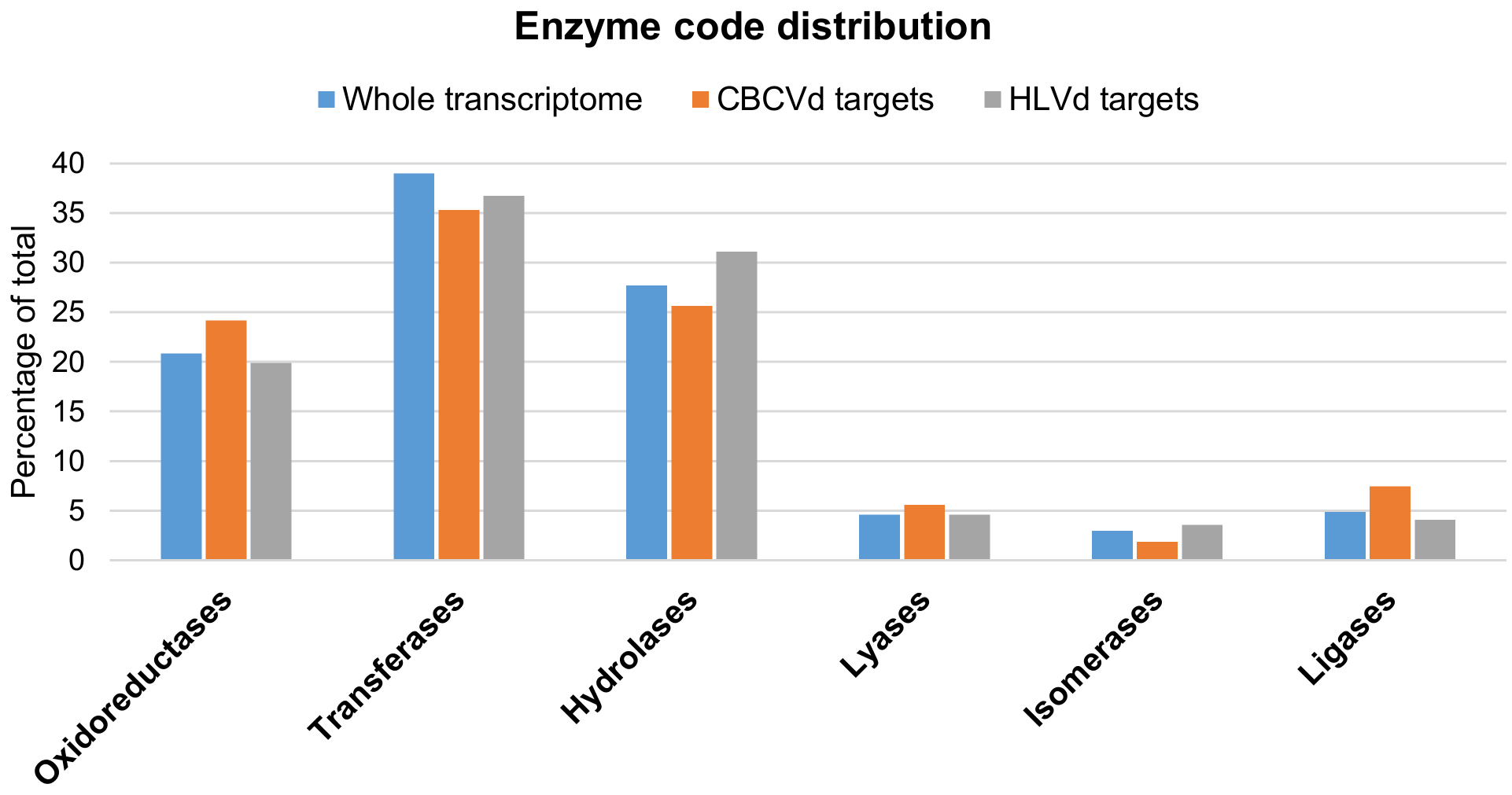

Supplement: S4 Fig — (TIFF) [file pone.0184528.s004.tiff]
