# Supplementary material for: Development of hop transcriptome to support research into host-viroid interactions
Source: PLoS One. 2017 Sep 8;12(9):e0184528. doi: 10.1371/journal.pone.0184528 (PMC5590963; doi:10.1371/journal.pone.0184528)
Supplement: S2 Table — (DOCX) [file pone.0184528.s006.docx]

**S2 Table. Developed primer pairs and PR primers used in RT-qPCR analysis and their PCR efficiencies.**

| **No.** | **Transcript Sequence /PR genes** | **Annotated gene abbreviation** | **Primer Sequence 5’-3’** | **qPCR efficiency [%]** |
| --- | --- | --- | --- | --- |
| 1 | contig_30192 | PLA1 | FOR: GTACCCGACCCAGCTCGAT REV: GCGACGTCGTCTGTTGGTT | 91.29 |
| 2 | contig_316 | LOX | FOR: TGCATCGCACTAAGTTGCCTAT REV: TGTTCACCAGCTCGTCAGTCA | 93.93 |
| 3 | contig_51543 | GA2ox | FOR: CAGCCGAGAAGCCTCGTCTA REV: TAAGTCTACTATCCCCAAGGCGTAA | 96.05 |
| 4 | contig_24780 | JAR1 | FOR: CTGTGCCGAGTGAAAGGTACAA; TCTCCTCACTCAACAACCAATCA | 102.96 |
| 5 | contig_4643 | DCL3a | FOR: GAGCTAGCATCTGAGAGCTGAAAA REV: AATACCGTCATTGATGCATCGA | 98.49 |
| 6 | contig_2112 | DCL1 | FOR: CGTAGTGGCGCCTCCAGTT REV: GGCTGCGAAACCAAAAGC | 93.93 |
| 7 | contig_29806 | COR413PM2 | FOR: AAGCAGGTAACAGCCGATGAG REV: CTGGTGGTGGCTCCAAGCT | 98.93 |
| 8 | contig_44350 | GATA | FOR: CTCCGCCGAGCTCTCTGTT REV: GGAATTAACGGGAAGCTGAAAA | 106.26 |
| 9 | contig_7166 | ACBD5 | FOR: GTCATCGAGCAGTCTGCCATT REV: TTTCCGCCGACAAGGAGTAC | 104.86 |
| 10 | contig_8499 | UP1 | FOR: TCTGCGACCAATCAGAACTGA REV: CTAGGCTGCCCACCATCTTC | 98.05 |
| 11 | contig_11357 | MBF1c | FOR: CTTCATCGAGCTTCCTCACGTT REV: CCGCTGTTCAGACCATCAAGA | 103.85 |
| 12 | contig_20309 | Ppol | FOR: CTCGATGCTTAGATCTCGGACAT REV: CTCGAAAGCTGGATTCATAAGGAA | 103.24 |
| 13 | contig_71362 | UP2 | FOR: GGAATCGAGGAAAACCCAGAT REV: CCTTCCTGTCAGGCCTTGAA | 106.03 |
| 14 | contig_18517 | UP3 | FOR: TTACTTTGAATAATGACGAGTGCTTGT REV: AGATCAATTAGCTGGGAGGGAAT | 101.88 |
| 15 | contig_21090 | AP3 | FOR: TGTTAAGGAGCACCTGTGACTCA REV: TTTCCGGTTGGCGGAGTAG | 98.31 |
| 16 | contig_24148 | SF3B | FOR: TCGCCCTGCTTGAATCAGA REV: GGAAGCTCAAGGTCCACCATT | 103.67 |
| 17 | contig_10708 | TBL24 | FOR: CTTGGCAAGAGCAACCCTTT REV: AGCCAGTTTGTTGCCTCAAAA | 97.89 |
| 18 | PR-1 |  | FOR: GAAGGTACCCTTATTGTTGTTGCA REV: GTTTGCGGGCACTACACTCA | 96.36 |
| 19 | PR-2 |  | FOR: TCCAAACTCAGATCTCCAAAAGC REV: CTCACACTTGGCCAGAAATTCA | 99.34 |
| 20 | PR-5 |  | FOR: AAACCAATTCAACAACCGTGACT REV: CACCCTCCCGTAGTGGGACTA | 100.66 |
| 21 | PR-3-EX |  | FOR: CACGGTCAAAAGTAGGATTGGAT REV: GCGAAAGGAGTTTGATTGTAGCA | 104.31 |
| 22 | PR-3-VAC |  | FOR: TGTGCTGCAGCCAATTCG REV: CACTTGGTTTGCATTGGCTTT | 98.26 |
